# Marine Compound Xyloketal B as a Potential Drug Development Target for Neuroprotection

**DOI:** 10.3390/md16120516

**Published:** 2018-12-19

**Authors:** Haifan Gong, Zhengwei Luo, Wenliang Chen, Zhong-Ping Feng, Guan-Lei Wang, Hong-Shuo Sun

**Affiliations:** 1 Department of Surgery, Faculty of Medicine, University of Toronto, Toronto, ON M5S 1A8, Canada; ann.gong@mail.utoronto.ca (H.G.); zhengwei.luo@mail.utoronto.ca (Z.L.); wenliang.chen@utoronto.ca (W.C.); 2 Department of Physiology, Faculty of Medicine, University of Toronto, Toronto, ON M5S 1A8, Canada; zp.feng@utoronto.ca; 3 Department of Pharmacology, Zhongshan School of Medicine, Sun Yat-Sen University, Guangzhou 510080, China; 4 Key Laboratory of Functional Molecules from Oceanic Microorganisms (Sun Yat-Sen University), Department of Education of Guangdong Province, Guangzhou 510080, China; 5 Department of Pharmacology and Toxicology, Faculty of Medicine, University of Toronto, Toronto, ON M5S 1A8, Canada; 6 Institute of Medical Science, Faculty of Medicine, University of Toronto, Toronto, ON M5S 1A8, Canada

**Keywords:** marine compound, xyloketal B, neuroprotection, antioxidative, drug development

## Abstract

Xyloketal B is a natural compound isolated from the mangrove fungus, *Xylaria* sp. in the South China Sea. In the past decade, studies have shown that xyloketal B exhibits anti-oxidative, anti-inflammatory, and anti-apoptotic abilities and may serve as a treatment for ischemic stroke. Xyloketal B has been shown to interact with both neurons and residential microglial cells and regulate a number of proteins involved in the apoptotic events during ischemia. Such mechanisms include inhibition of specific NADPH oxidase subunits, upregulation of HO-1, increase of Bcl-1/Bax ratio, and downregulation of TLR4 receptor. Both *in vitro* and *in vivo* stroke models have validated its potential in preventing ischemia-induced neuronal cell death. This review summarizes our current understanding of the effects of xyloketal B in ischemic conditions. As stroke ranks second in the causes of mortality worldwide and still lacks effective treatment, it is necessary to seek novel therapeutic options. Understanding the role of xyloketal B in ischemic stroke could reveal a new aspect of stroke treatment.

## 1. Introduction

Despite significant improvement in prevention strategies and quality of hospital care over the past few decades, stroke still ranks second in the causes of death worldwide [1,2]. It is also the number one cause of adult long-term disability and immobility in the United States [1]. As effective treatments for stroke remain obscure, mortality rate stays high at approximately one-third of patients, and a further one-third suffer from residual disability [3]. Tissue-type plasminogen activator (tPA) is currently the only FDA-approved treatment option for stroke. It has an extremely brief therapeutic window of 3–4 h after the onset of stroke and may leave detrimental damage to the blood-brain barrier (BBB) if administered beyond 3 h [4,5]. Research in novel drug targets for stroke treatment has been on the rise as stroke is not only a medical issue but also a burden on economic development internationally.

Around 80–90% of all strokes are ischemic [4,6,7]. Cerebral ischemia is caused by the obstruction of cerebral blood vessels which leads to reduced blood flow and energy supply to the affected brain tissues. Consequently, cells lose their ability to maintain ionic balance and release an excessive amount of excitatory neurotransmitters, namely glutamate, into the extracellular matrix [7,8,9]. Over-activated glutamate receptors, specifically the *N*-Methyl-D-aspartate (NMDA) and the α-amino-3-hydroxy-5-methyl-4-isoxazolepropionic acid (AMPA) receptors, contribute to the intracellular overload of ions, especially Ca^2+^. This process is referred to as excitotoxicity [8,9]. Not only that excitotoxicity leads to lethal brain edema, excessive Ca^2+^ also triggers a number of signaling pathways that contribute to neuronal cell death [8,9]. Cells experience an increase in proteolytic enzymes and reactive oxygen species (ROS), including hydrogen peroxide and superoxide anion, that overcome the cell’s anti-oxidative defense mechanism and result in oxidative stress. Combined with a number of other events, including the release of cytochrome c due to mitochondrial impairment, the cell induces apoptosis minutes after the onset of stroke [8,9]. Drugs targeting NMDA receptor activities have been experiencing unsuccessful clinical trials due to the crucial role of NMDA receptors in normal neuronal activities [10,11]. The development of new drugs targeting non-traditional pathways is still under investigation.

Natural compounds are favored by researchers in drug discovery for human diseases as they have higher efficacy due to co-evolution with their biological targets [12]. Natural therapeutics derived from terrestrial organisms are currently in the lead in pharmaceutical research, however, marine organisms have become a more popular source for novel drugs in the past decades. As a result of their unique habitats that often require adaptation to challenging physical and chemical properties, including oxygen and ion concentrations, temperature, and pressure [13,14], marine lives are able to develop new natural compounds capable of combating difficult conditions that humans experience in abnormal states. Statistics have shown that the number of marine-sourced natural molecules that demonstrate significant bioactivity, such as potential anti-tumor actions, is ten times higher than the terrestrial-sourced molecules [12]. As a result, the number of reports on new potential therapeutics isolated from marine organisms has been rapidly increasing.

Starting from 2001, Lin and colleagues isolated a class of natural compounds from the mangrove fungus, *Xylaria* sp. (no. 2509) from the South China Sea coast and named xyloketals A-G [15,16]. Among these compounds, xyloketal B exhibits a hydroxy-phenol radical which may serve as a scavenger for free oxygen radicals and thus prevents ischemic neuronal cell damage through glutamate-independent mechanisms [17,18]. Over the past decade, xyloketal B has been proven to reduce cell and tissue damage after ischemic conditions in both *in vitro* and *in vivo* models. It may prevent neuronal death through: (1) Decreasing free radical level by inhibiting ROS- and reactive nitrogen species (RNS)-producing enzymes and enhancing anti-oxidative enzymes [17,18,19,20,21]; (2) inhibiting mitochondrial damage and the initiation of apoptosis by increasing the expression of anti-apoptotic proteins [17,18,20]; (3) elevating the expression of the anti-oxidative protein HO-1 through Nrf2/ARE pathway [21]; (4) blocking excessive calcium entry during ischemia [20]; and (5) reducing the level of inflammatory cytokines via decreasing TLR4 and NF-κB expression in residential microglial cells [19].

## 2. The Anti-Oxidative and Anti-Apoptotic Effects of Xyloketal B in Endothelial Cells

In 2009, Chen and colleagues were the first to examine the protective effect of xyloketal B on oxidized low-density lipoprotein (oxLDL)-induced cell injury *in vitro* [17]. oxLDL in the blood triggers a series of pathological events in the endothelial lining of the blood vessel, including the activation of transcription factor NF-κB, which in turn induces the production of ROS and other inflammatory factors [22] similar to that in neurons during ischemia. Meanwhile, the production of nitric oxide (NO) is decreased which attenuates its ROS-scavenging and neuroprotective activities at the physiological level [23]. By reacting with NO, excessive ROS are also able to produce peroxynitrite (ONOO^−^), a powerful oxidant [17]. As a result, damaged endothelial cells elicit the formation of atherosclerotic plaques and ultimately lead to atherosclerosis, the most common cause of cerebral ischemia.

Chen and colleagues (2009) revealed the earliest evidence that demonstrates xyloketal B’s cytoprotective ability. An *in vitro* model utilizing human umbilical vein endothelial cells (HUVECs) was used to mimic oxLDL-induced endothelial injury [17]. While incubating HUVECs with oxLDL was observed to cause cell morphological changes and decreases in cell viability, xyloketal B was able to significantly revert these effects in a dose-dependent manner from 0.3 to 40 µM [17]. To understand xyloketal B’s anti-apoptotic mechanism in greater depth, Chen and colleagues evaluated its effect on oxLDL-induced NADPH oxidase activity. Nicotinamide adenine dinucleotide phosphate (NADPH) oxidase has a key role in intracellular ROS production and its activity has been observed to be increased in atherosclerotic arterial cells [24]. A higher mRNA expression level has also been observed in gp91*phox*, one of the subunits of the NADPH oxidase complex, in oxLDL-induced atherosclerotic human endothelial cells at the same time as increased NADPH oxidase activity [25]. Chemiluminescence results from Chen and colleagues’ study showed that pre-treatment of xyloketal B was able to significantly lower both oxLDL-induced superoxide anion production and the mRNA expression of NADPH oxidase subunits gp91*phox* and p47*phox* [17]. These findings indicate that xyloketal B inhibits ROS production via inhibiting NADPH oxidase activity by decreasing the mRNA expression of its subunits. Similarly, the release of NO was promoted by xyloketal B, which restores the balance between ROS and NO and in turn inhibits the production of peroxynitrite following oxLDL-injury [17]. In 2015, a study has also reported a significant reduction in H_2_O_2_-induced HUVEC injury by two derivatives of xyloketal B [26]. All of these findings have proven the anti-oxidative effect of xyloketal B *in vitro*.

Lastly, western blot results have proven xyloketal B’s ability to restore the expression of Bcl-2 that was decreased by oxLDL incubation [17]. Bcl-2 is a crucial anti-apoptotic protein that plays a key role in cell death in both epithelial cell injury and cerebral ischemia via the mitochondrial-dependent apoptotic pathway [27,28]. As a result, xyloketal B may be not only effective in oxLDL-induced cell death, but also neuronal cell death in ischemic conditions through regulating Bcl-2 expression. All these findings have brought xyloketal B’s protective effects to attention and set a stepping stone for future research of possible applications of xyloketal B in the nervous system.

Similar findings of xyloketal B’s endothelial-protective effects were reported by Zhao and colleagues in 2015 with an apolipoprotein E knockout (ApoE^−/−^) mouse model [29]. In endothelial cells with atherosclerotic condition, xyloketal B (20 mg/kg) was observed to reduce atherosclerotic plaque formation and lesion. It was found to promote phosphorylation of endothelial NO synthase (eNOS) at Ser-1177 and restore ROS and NO balance [29]. This once again confirmed the anti-oxidative and anti-inflammatory capabilities of xyloketal B.

## 3. The ROS-Scavenging and Mitochondrial-Protective Abilities of Xyloketal B in Neurons

Later in 2009, Zhao and colleagues tested the neuroprotective potential of xyloketal B [18]. First, they demonstrated the ability of xyloketal B to scavenge free oxygen radicals using the 2,2-Diphenyl-1-picrylhydrazyl (DPPH) assay, confirming its anti-oxidative activity. In order to observe xyloketal B’s effects on neuronal cells, PC12 neuronal cell line was exposed to oxygen and glucose deprivation (OGD), a widely used model to mimic ischemic conditions in the brain [30]. While the MTT (3-(4, 5-dimethylthiazolyl-2)-2, 5-diphenyltetrazolium bromide) assay detected a significant reduction in the number of viable cells after OGD insult, pretreatment of xyloketal B yielded higher cell viability concentration-dependently from 12.5 to 200 µM [18]. Using DAPI (4′,6-diamidino-2-phenylindole) staining, a significantly lesser extent of nuclear abnormality was also observed in the xyloketal B-treated group compared to those in the vehicle controls [18]. The level of protein carbonylation was used as another indicator for oxidative stress as oxidized protein side chains contain carbonyl groups [31]. Oxiblot results showed no significant difference in the level of carbonylation between the control and xyloketal B-treated groups, while the vehicle group displayed a significantly higher level [18]. All of which have first established xyloketal B’s anti-apoptotic therapeutic potential for stroke in a neuronal setting.

The experiment then focused on xyloketal B’s influence on mitochondrial function during OGD insult [18]. The disturbance of cellular respiration due to a glucose shortage during ischemia results in the accumulation of NADH in the mitochondria. Excessive reduction by NADH over-produces ROS, which further leads to mitochondrial damage [32]. Through events including the breakdown of mitochondrial membrane and decreases in mitochondrial membrane potential (MMP), cytochrome c is released into the cytoplasm which onsets apoptosis [32,33]. In this study, mitochondrial ROS production was detected to be significantly increased after OGD by MitoSOX assay [18]. Xyloketal B-treated group, on the other hand, had the MitoSOX signal intensity decreased by 27%. Using fluorescence microscopy, Zhao and colleagues observed a significant increase in the number of fragmented mitochondria post-OGD as a result of oxidative stress, which was reduced by the treatment of xyloketal B. Consistently, xyloketal B was also found to bring the increased level of Drp1 back to normal level after OGD [18]. Drp1 is a protein that promotes excessive mitochondrial fission when the pro-apoptotic protein Bax is over-expressed during OGD. Drp1-dependent mitochondrial fragmentation also leads to the release of cytochrome c and apoptosis [34,35]. Moreover, OGD-induced decreases in MMP was reversed by xyloketal B treatment as well. These experiments have concluded that the mitochondrion is the potential target in the anti-apoptotic effect of xyloketal B in neurons.

## 4. Xyloketal B Promotes Expression of HO-1 by Regulating the Upstream Signaling Pathway

Xyloketal B has been shown to regulate the expression of a number of anti-stress compounds in the cell, including Bcl-2 as previously mentioned and a crucial anti-oxidant glutathione [36]. To further elaborate on its anti-oxidative capability, Li and colleagues (2013) investigated xyloketal B’s effect on gene induction of a stress protein, heme oxygenase-1 (HO-1) [21]. HO-1 is a protective enzyme that is responsible for converting heme to free iron, biliverdin, and carbon monoxide (CO). Biliverdin is readily further converted to bilirubin in the body and both have been shown to have anti-oxidative abilities [33,37]. CO has been confirmed to decrease the expression of pro-inflammatory factors and also upregulate heat shock protein 70 (Hsp70) to prevent cytokine-induced apoptosis [33]. The activity of NADPH oxidase can be suppressed by HO-1 as well [38]. Since HO-1 protects the cell against a variety of stress conditions, including ischemia, its expression is regulated by a number of signaling pathways, such as nuclear factor-erythroid 2-related factor 2 (Nrf2), phosphatidyl inositol 3-kinase (PI3k)/protein kinase B (Akt), and mitogen-activated protein kinases (MAPKs) pathways [21,33].

Li and colleagues (2013) found that pretreatment of 20 µM xyloketal B reduced the number of apoptotic cells and morphological damage after angiotensin II (AngII)-induced injury in HUVECs [21]. Similar to previous studies, AngII-induced ROS overload was attenuated in xyloketal B-treated groups as well. The anti-oxidative ability of xyloketal B was not only once again confirmed *in vitro*, it demonstrated the same effect in the embryo respiratory burst model in zebrafish. In PMA-induced embryos which over-produce ROS, pre-incubation of xyloketal B was able to decrease the NADPH oxidase activity in a concentration-dependent manner. Additionally, an HO-1-specific inhibitor, SnPP, averted this cytoprotective effect of xyloketal B in both *in vitro* and *in vivo* experiments. This indicates HO-1 as the target in the anti-oxidative process of xyloketal B.

Next, Li and colleagues examined the underlying mechanism of the regulation of HO-1 activity by xyloketal B. When incubated with xyloketal B, HUVACs displayed significantly increased HO-1 mRNA levels up to 24 h and progressively increased HO-1 expression levels up to 36 h. Remarkably, the nuclear accumulation of Nrf2 and its binding activity to the antioxidant response element (ARE) were found to be higher in the treatment group compared to the control in HUVECs.

Furthermore, PI3k/Akt and MAPKs are upstream regulators of Nrf2 nuclear translocation for an HO-1-inducing signal [33]. In this study, xyloketal B was found to have phosphorylating capabilities of Akt and ERK1/2 [21]. Xyloketal B-induced HO-1/Nrf2 induction was attenuated when PI3k/Akt and ERK1/2 (MAPKs) were inhibited. These findings suggest that these are the upstream pathways responsible for the increased HO-1 expression by xyloketal B. Together, this article revealed that HO-1 induction through Nrf2/ARE and other pathways is responsible for the anti-oxidative effect of xyloketal B.

Alternatively, Zhou and colleagues in 2018 have reported that a xyloketal B derivative increased the expression of Hsp70 by upregulating the transcriptional activity of heat shock transcription factor-1 (HSF-1) in the laboratory strain (N2) *Caenorhabditis elegans* [39]. This indicates that xyloketal B may interact with multiple pathways in its anti-inflammatory response.

## 5. Xyloketal B’s Neuroprotective Effect in Neonatal Hypoxic-Ischemic Brain Injury Model

In 2015, Xiao and colleagues for the first time investigated the ability of xyloketal B to protect against ischemic brain injury with both *in vitro* and *in vivo* models [20]. Neonatal hypoxic-ischemic (HI) brain injury is caused by oxygen deprivation during birth which leads to severe brain damage in newborns, especially in regions with high metabolic activity [40]. The resulting condition is named hypoxic-ischemic encephalopathy (HIE), which results in perinatal mortality or a wide range of cognitive and motor disabilities in children [41]. Regardless of HI injury’s high incidence of 0.5–1 per 1000 births in developed countries, there still lacks effective treatments, especially for preterm infants [42].

In the first part of this study, the protective effect of xyloketal B was demonstrated with OGD-induced neuronal death in mouse embryonic primary cortical neuron culture [20]. Propidium iodide (PI) staining showed increased cell viability with the pretreatment of xyloketal B compared to the vehicle group after OGD. To investigate the underlying mechanism of xyloketal B’s suppression on OGD-induced neuronal death, Xiao and colleagues observed xyloketal B’s effects on excessive calcium influx during ischemia using fura-2 calcium imaging. The data suggested that the neuroprotective capacity of xyloketal B was partially due to its ability to block calcium entry [20].

Xiao and colleagues then proceeded to use a neonatal hypoxic-ischemic brain injury (HI) model to further evaluate xyloketal B *in vivo*. Right common carotid artery occlusion was performed on post-natal day-7 (P7) CD1 mouse pups, followed by 100 min of hypoxia with 8% oxygen. The pretreatment of xyloketal B (5 mg/kg, i.p.) 30 min before the onset of HI remarkably reduced the infarct volume in the ipsilateral hemisphere compared to the vehicle group both 24 h and 7 days post-HI [20]. When body weight was compared between the groups on post-HI day 7, the treatment group had no significant difference with the sham group, while the vehicle group had significantly less weight gain. When the neurobehavioral tests including geotaxis reflex, cliff avoidance reaction, and grip test were performed, animals in the treatment group were observed to have more optimal performance than the vehicle group [20]. These findings have proven that xyloketal B is effective in improving neuronal viability and recovery in terms of weight gain and sensorimotor functions. Furthermore, the anti-apoptotic effect of xyloketal B was once again demonstrated by its ability to reduce the amount of DNA fragmentation in the penumbral area of the ipsilateral hemisphere shown by TUNEL (terminal deoxynucleotidyl transferase dUTP nick end labeling) assay.

When investigating the action mechanism of xyloketal B at the molecular level, the levels of proteins including Bax, cleaved caspase-3, and Bcl-2 were examined. Bax is a pro-apoptotic effector protein that inhibits anti-apoptotic proteins including Bcl-2 upon the activation by death stimuli [27]. As a result, the Bcl-2/Bax ratio is recognized as an indicator for the apoptotic susceptibility of a cell [43,44]. A low Bcl-2/Bax ratio promotes Drp1-dependent mitochondrial fission, as previously described, and releases cytochrome c into the cytoplasm that cleaves caspase-3 to its activated form [34,35,43]. Although Xiao and colleagues observed a significant surge in cleaved caspase-3 level and a low Bcl-2/Bax ratio 24 h after HI in the neonatal brains without treatment, xyloketal B was able to bring these values to the normal ranges where there was no significant difference from the sham group [20]. These pieces of evidence indicate that xyloketal B prevents the cell from executing apoptosis by modulating Bcl-2 and Bax levels. Altogether, this study successfully demonstrated the anti-apoptotic and neuro-recovery potential of xyloketal B post-ischemia *in vivo* using a neonatal HI model.

## 6. The Neuroprotective Potential of Xyloketal B in Adult Ischemia Model

To completely unravel the potential role xyloketal B may play in cerebral ischemia, Pan and colleagues (2017) examined treatment outcomes of xyloketal B after ischemia using a transient middle cerebral artery occlusion (tMCAO) model in adult male C57 mice [19]. tMCAO mimics ischemic conditions by restricting blood flow in the cerebral artery using an intraluminal suture for a period of 2 h. To evaluate xyloketal B’s preventive potentials, three intraperitoneal injections of xyloketal B of 12.5, 25, and 50 mg/kg were administered at 48 h, 24 h, and 30 min before tMCAO. As triphenyltetrazolium chloride (TTC) staining of the ipsilateral hemisphere 24 h after the occlusion displays a relative infarction volume of approximately 26%, the animal group that received multiple injections of xyloketal B pretreatment has a significantly reduced infarction volume in a concentration-dependent manner [19]. Remarkably, the infarction volume was also significantly reduced when a single dose (50 mg/kg, i.p.) was administered at 0, 1, or 2 h after the occlusion, demonstrating the potential therapeutic time window for xyloketal B. Among the results, a single-dose injected immediately after tMCAO (0 h) displayed the smallest area of infarction and was used in the subsequent experiments.

A four-tiered grading system developed by Benderson et al. (1986) was then utilized to evaluate post-ischemic neuromotor deficiency, which positively correlates with the size of the lesion in the frontal cortex and striatum [45]. In this study, the neurological deficiency scores after ischemic insult with xyloketal B treatment were improved comparable to the positive control group, while the vehicle group displayed higher deficiency [19]. These results indicate that xyloketal B exhibits neuroprotective capability in terms of reducing brain damage and the extent of consequential neurological deficiency after ischemia.

To further investigate the underlying mechanism of the neuroprotective effects of xyloketal B in this tMCAO model, Pan and colleagues evaluated the change in the post-ischemic production of free radicals with xyloketal B treatment [19]. As previously mentioned, both ROS and RNS including NO are produced in excessive amounts during ischemia that lead to severe cellular damage [46]. Dihydroethidium (DHE)-stained brain tissues showed a significant reduction in the ROS level in the xyloketal B-treated group compared to the vehicle group. The expression of an important RNS-producing enzyme, inducible NO synthase (iNOS), was also significantly lowered by xyloketal B [19]. At the same time, the expression of an anti-oxidative enzyme, manganese superoxide dismutase (MnSOD), was critically elevated by xyloketal B treatment. MnSOD converts superoxide anions to hydrogen peroxide and molecular oxygen, which in turn prevents oxidative damage of the cell [47]. This evidence once again confirms the anti-oxidative ability of xyloketal B in ischemic conditions.

As previously discussed, oxygen and energy shortage results in the onset of apoptosis during ischemia. Following the brain injury, residential microglial cells are activated and trigger inflammation through toll-like receptor 4 (TLR4) and the subsequent intracellular signaling pathways. As a result, nuclear factor-κB (NF-κB) is released, which in turn initiates the expression of inflammatory cytokines including TNF-α, IL-1β, IL-6, and IFN-γ [48,49]. The blood-brain barrier is subsequently damaged to allow leukocyte infiltration/diapedesis, resulting in tissue damage. The inhibition of cytokine release is suggested to protect BBB integrity [48] and prevent neuronal cell death. In this study, Pan and colleagues evaluated the mRNA levels of TNF-α, IL-1β, IL-6, and IFN-γ in the brain tissue after tMCAO with quantitative real-time polymerase chain reaction (PCR). The xyloketal B-treated tissues were observed to have significantly lower levels of the inflammatory cytokines than the vehicle group [19]. At the same time, Evans blue extravasation was performed to examine BBB permeability post-ischemia. The data indicate that xyloketal B successfully attenuated BBB disruption by ischemia, which correlated with the qPCR data [19]. Furthermore, the expression level of TLR4 and NF-κB were found to be higher after tMCAO, but both Western blot and immunohistochemistry staining results supported xyloketal B’s ability to attenuate the excessive expression of both proteins [19]. The translocation of NF-κB from the cytoplasm to the nucleus was also disturbed in xyloketal B-treated samples, meaning that excessive production of inflammatory cytokines was inhibited [19]. Altogether, these findings demonstrate xyloketal B’s ability to prevent neuronal cell death and deterioration of BBB integrity after ischemia.

In the most recent xyloketal B study, Zhao and colleagues (2018) have proven xyloketal B’s ability in the reduction of blood pressure and relaxation of aortic rings in 2K2C renovascular hypertensive rats [50]. As hypertension is one of the major risk factors for ischemic stroke, these findings further validate the beneficial effects of xyloketal B in cardiovascular patients.

## 7. Conclusions

**Figure 1:**
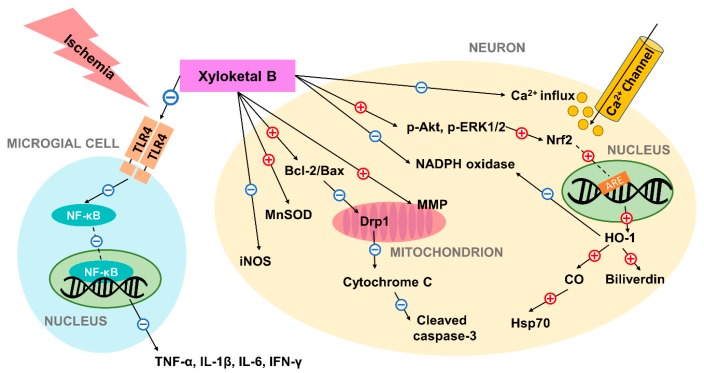
Schematic diagram of the neuroprotective mechanism of action of xyloketal B in ischemic condition. Previous studies have suggested that xyloketal B may prevent ischemic neuronal death via (1) decreasing ROS and RNS levels through regulation of enzymes including iNOS, MnSOD, NADPH oxidase; (2) controlling Bcl-2/Bax ratio and preventing subsequent mitochondrial damage and apoptosis; (3) increasing the expression of the stress protein HO-1 through Nrf2/ARE pathway, and thus producing cytoprotective compounds; (4) reducing excessive calcium influx; and (5) inhibiting expression of pro-inflammatory cytokines by decreasing TLR4 and NF-κB expression in residential microglial cells. In the figure, the red plus sign (+) indicates stimulation while the blue subtraction sign (−) indicates inhibition. Dashed lines are used to represent nuclear translocation. Abbreviations: ARE: antioxidant response element; HO-1: heme oxygenase-1; Hsp70: heat shock protein 70; iNOS: inducible NO synthase; MnSOD: manganese superoxide dismutase; MMP: mitochondrial membrane potential; TLR4: toll-like receptor 4; NF-κB: nuclear factor-κB; ROS: reactive oxygen species; RNS: reactive nitrogen species.

Evidence showed that the marine compound xyloketal B has anti-oxidative and neuroprotective effects in preventing ischemic neuronal cell death mediating few different signaling pathways, concluded in Figure 1. Studies have compiled considerable indications of its anti-oxidative and anti-apoptotic potentials in ischemic conditions both *in vitro* and *in vivo*, which are summarized in Table 1. Xyloketal B is capable of regulating expression of a number of proteins that participate in post-ischemic apoptotic events. Further investigations of the full therapeutic mechanism of action are expected. Regardless, xyloketal B’s neuroprotective effects may allow it to serve as a novel treatment alternative for ischemic stroke and improve stroke outcome.

## Figures and Tables

**Table 1:** Summary of Xyloketal B-Related Experiments Discussed.

*In Vitro*/*In Vivo*	Cells/Animals	Model	Dose/Concentration	Major Findings
*In vitro*	HUVECs	OxLDL induced oxidative injury	0.3 to 40 μM	Cytoprotective effect; Decreased ROS generation; Increased NO generation [17]
*In vitro*	PC12	OGD model of ischemic stroke	12.5 to 800 μM	ROS-scavenging and mitochondrial-protective abilities [18]
Both	PC12 and *C. elegans*	MPP+-induced neurotoxicity	25 to 250 μM	Reduction of ROS generation and restoration of anti-oxidant glutathione level [36]
Both	HUVECs and zebrafish	AngII-Induced HUVEC apoptosis and PMA-induced respiratory burst of zebrafish embryos	0.2 to 80 μM	Increase in HO-1 expression through PI3K/Akt signaling pathway [21]
Both	Mouse primary cortical cells and CD1 mice	OGD in cortical cells and neonatal hypoxic-ischemic brain injury	10 to 100 μM *in vitro*; 5 mg/kg body weight *in vivo*	Neuro-protection in neonatal ischemic brain injury [20]
*In vitro*	HUVECs	H_2_O_2_-induced HUVEC injury	20 and 25 μM	Xyloketal B and its two derivatives inhibited H_2_O_2_-induced HUVEC injury [26]
Both	HUVECs and apolipoprotein E-deficient mice	High-fat diet-induced atherosclerosis	10 to 80 μM *in vitro*; 7, 14, and 28 mg/kg/day *in vivo*	Reduction of aortic atherosclerotic lesion area and improved endothelia function via increasing NO generation [29]
*In vivo*	C57 mice	Transient middle cerebral artery occlusion (tMCAO)	50 mg/kg body weight	Pretreatment reduced infarction volume dose-dependently [19]
*In vivo*	*C. elegans*	Heat stress	100 μM	Xyl-B derivative increased the expression of Hsp70 by upregulating HSF1 activity [39]
*In vivo*	Sprague Dawley rats	Two-kidney, two-clip renovascular hypertensive model	20 μM in aortic ring function; 20 mg/kg body weight	Reduced blood pressure and enhanced relaxation of aortic rings in 2K2C renovascular hypertensive rats [50]

Abbreviations: HUVECs: human umbilical vein endothelial cell; oxLDL: oxidized low-density lipoprotein; ROS: reactive oxygen species; NO: nitrogen oxide; OGD: oxygen and glucose deprivation; *C. elegans*: *Caenorhabditis elegans*; MPP+: 1-methyl-4-phenylpyridinium; AngII: angiotensin II; PMA: phorbol 12-myristate 13-acetate; HO-1: Heme oxygenase 1; Hsp70: heat shock protein 70; HSF1: heat shock factor 1; 2K2C: 2-kidney, 2 clip.
